# Synthesis of Antimicrobial Chitosan-Silver Nanoparticles Mediated by Reusable Chitosan Fungal Beads

**DOI:** 10.3390/ijms24032318

**Published:** 2023-01-24

**Authors:** Edward Hermosilla, Marcela Díaz, Joelis Vera, María José Contreras, Karla Leal, Rodrigo Salazar, Leticia Barrientos, Gonzalo Tortella, Olga Rubilar

**Affiliations:** 1 Chemical Engineering Department, Universidad de La Frontera, Temuco 4811230, Chile; 2 Biotechnological Research Center Applied to the Environment (CIBAMA-BIOREN), Universidad de La Frontera, Temuco 4811230, Chile; 3 Programa de Doctorado en Ciencias de la Ingeniería, Universidad de La Frontera, Temuco 4811230, Chile; 4 Extreme Environments Biotechnology Lab, Center of Excellence in Translational Medicine, Universidad de La Frontera, Av. Alemania 0458, Temuco 4811230, Chile; 5 Scientific and Technological Bioresource Nucleus (BIOREN), Universidad de La Frontera, Temuco 4811230, Chile; 6 Center of Excellence in Traslational Medicine (CEMT), Universidad de La Frontera, Av. Alemania 0458, Temuco 4811230, Chile

**Keywords:** nanoparticle synthesis, chitosan-silver nanoparticles, reusable fungal biomass, antimicrobial activity

## Abstract

Nanoparticles, especially silver nanoparticles (Ag NPs), have gained significant attention in recent years as potential alternatives to traditional antibiotics for treating infectious diseases due to their ability to inhibit the growth of microorganisms effectively. Ag NPs can be synthesized using fungi extract, but the method is not practical for large-scale production due to time and biomass limitations. In this study, we explore the use of chitosan to encapsulate the mycelia of the white-rot fungus *Stereum hirsutum* and form chitosan fungal beads for use in multiple extractions and nanoparticle synthesis. The resulting nanoparticles were characterized using various techniques, including UV-vis spectrophotometry, transmission electron microscopy, dynamic light scattering, and X-ray diffraction analysis. The analysis revealed that the synthesized nanoparticles were composed of chitosan-silver nanoparticles (CS-Ag NPs) with a size of 25 nm. The chitosan fungal beads were reused in three extractions and nanoparticle synthesis before they lost their ability to produce CS-Ag NPs. The CS-Ag NPs showed potent antimicrobial activity against phytopathogenic and human pathogenic microorganisms, including *Pseudomonas syringae*, *Escherichia coli*, *Staphylococcus aureus*, and *Candida albicans*, with minimum inhibitory concentrations of 1.5, 1.6, 3.1, and 4 µg/mL, respectively. The antimicrobial activity of CS-Ag NPs was from 2- to 40-fold higher than Ag NPs synthesized using an aqueous extract of unencapsulated fungal biomass. The CS-Ag NPs were most effective at a pH of five regarding the antimicrobial activity. These results suggest that the chitosan fungal beads may be a promising alternative for the sustainable and cost-effective synthesis of CS-Ag NPs with improved antimicrobial activity.

## 1. Introduction

The overuse of traditional antibiotic drugs has led to a rise in antibiotic resistance among pathogenic microorganisms, which poses a threat to human health and agriculture [1]. In order to address this problem, researchers are exploring new solutions and approaches, such as the synthesis of novel and efficient nanomaterials as alternatives to conventional antibiotics. Silver nanoparticles (Ag NPs) have emerged as a promising option in this field due to their powerful antimicrobial action against a wide range of pathogenic microorganisms, including fungi, yeast, and bacteria [2,3,4]. There are several approaches to synthesizing Ag NPs, each with its own advantages and disadvantages. Some standard synthesis methods include chemical reduction, physical vapor deposition, and template-assisted methods [5]. Chemical reduction methods involve the reduction of silver ions to silver atoms using a reducing agent, which can then form Ag NPs through a process known as nucleation. This method is simple, relatively easy to scale up [6], and produces Ag NPs with high stability and monodispersity, but it can generate significant amounts of chemical waste, which can be difficult to dispose of and has negative environmental impacts. Physical vapor deposition methods involve the deposition of silver atoms onto a substrate to form Ag NPs [5]. This method can produce high-quality Ag NPs with good stability and monodispersity, but it can be expensive and time-consuming. The use of green methods for Ag NP synthesis is becoming increasingly important as concerns about the environmental impacts of traditional synthesis techniques continue to grow [7]. These methods often involve using natural materials or biodegradable compounds as reducing agents and stabilizers, offering a sustainable and cost-effective alternative for producing high-quality Ag NPs with improved stability and bioactivity [8]. The green synthesis of Ag NPs using an extracellular extract of fungi is a simple, environmentally friendly, and cost-effective method. This method typically involves the production of fungal biomass, the separation of the biomass, the preparation of an aqueous extract, and the reduction of Ag+ to Ag^0^ at the nanoscale by the extract [9]. The aqueous extract of fungi contains a variety of extracellular metabolites, including anthraquinones, amino acids, nicotinamide adenine dinucleotide (NADH), polysaccharides, nitrate reductase, extracellular proteins, and other enzymes [9,10,11]. It is hypothesized that these metabolites have the potential to reduce and stabilize synthesized nanoparticles. However, the time required to obtain a sufficient fungal biomass and the inability to reuse the biomass for multiple syntheses are major drawbacks to the feasibility of large-scale Ag NP synthesis. One potential solution to these challenges is immobilizing a fungal biomass using a biocompatible polymer, such as chitosan [12]. Chitosan forms gels when interacting with divalent and polyvalent anions and has been used to encapsulate chemical compounds, enzymes, cells, and microorganisms [13,14,15,16]. The chitosan immobilization of fungi provides stability, a higher surface area, homogeneity, and easy separation of the chitosan fungal beads, allowing for the preparation of multiple aqueous extracts for Ag NP synthesis. In a previous work, we demonstrated the synthesis of Ag NPs with antimicrobial activity against *Pseudomonas syringae* using an aqueous extract of *Stereum hirsutum* biomass [17]. In this study, we explored the chitosan encapsulation of *S. hirsutum* mycelia to form chitosan fungal beads for its use in multiple cycles of aqueous extract preparation and subsequent nanoparticle synthesis. We also assessed the antibiotic activity of the synthesized nanoparticles against phytopathogenic and human pathogenic microorganisms.

## 2. Results and Discussion

**Figure 1:**
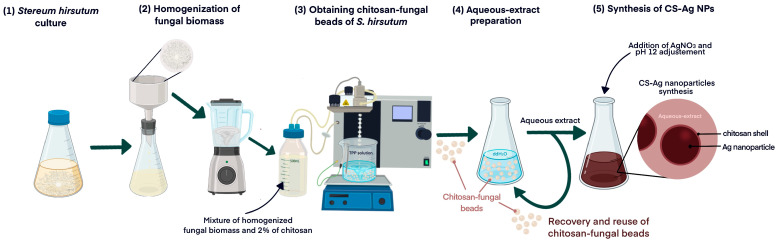
Diagram of the process of obtaining chitosan fungal beads using chitosan and the subsequent synthesis of CS-Ag NPs.

In a previous investigation, we described the successful synthesis of Ag NPs using an aqueous extract of the mycelia of the fungus *S. hirsutum* (CCCT22.02) [17]. Building on these findings, the current study aims to evaluate the synthesis of nanoparticles by utilizing a consecutive series of aqueous extracts derived from chitosan fungal beads. These beads are composed of the mycelia of *S. hirsutum* encapsulated within chitosan, which serve as a reusable source of compounds involved in the synthesis. The methodology employed in this study is outlined in the diagram presented in Figure 1.

### 2.1. Encapsulation of S. hirsutum in Chitosan Beads

**Figure 2:**
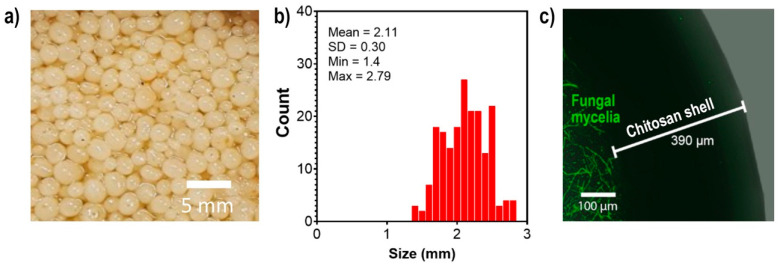
Image (**a**), size distribution (**b**), and CLSM image (**c**) of chitosan fungal beads of *S. hirsutum* for use in multiple extractions and nanoparticle synthesis.

The encapsulation of *S. hirsutum* using a 2% chitosan solution (low molecular weight) resulted in the formation of spherical chitosan fungal beads with a diameter of 2.11 mm, as shown in Figure 2b. Confocal laser scanning microscopy analysis showed that the fungal mycelia were located inside the beads, surrounded by a 390 µm layer of chitosan (Figure 2c). Chitosan has been shown to have the potential to encapsulate various agents, including enzymes, drugs, bacteria, and fungi [13,15,16]. This property of chitosan has been exploited for various purposes, including transport and bioremediation. The encapsulation of *S. hirsutum* using chitosan provides higher stability and homogeneity of mycelia, making it suitable for its reuse in the extract preparation and subsequent synthesis of Ag NPs.

### 2.2. Synthesis of Nanoparticles Using the Aqueous Extract of Chitosan Fungal Beads

The synthesis of nanoparticles using chitosan fungal beads and AgNO_3_ (3 mM) was evidenced by the color change from colorless to dark brown. No color change was observed in the synthesis reaction using the aqueous extract of chitosan beads without fungus. Therefore, the agents responsible for reducing Ag^+^ to Ag^0^ during the synthesis of nanoparticles were released by the fungal content found in the chitosan fungal beads. As previously reported, the extract obtained from the unencapsulated biomass of *S. hirtusum* contains various compounds with different molecular weights that can play both a reducing and stabilizing role in the synthesis of Ag NPs [17]. Cisternas et al. (2021) proposed that low molecular weight compounds such as hydroquinone could serve as a reducing agent in the synthesis of nanoparticles, while proteins containing L-cysteine residues may have dual roles as both reducing agents and stabilizers [4].

**Figure 3:**
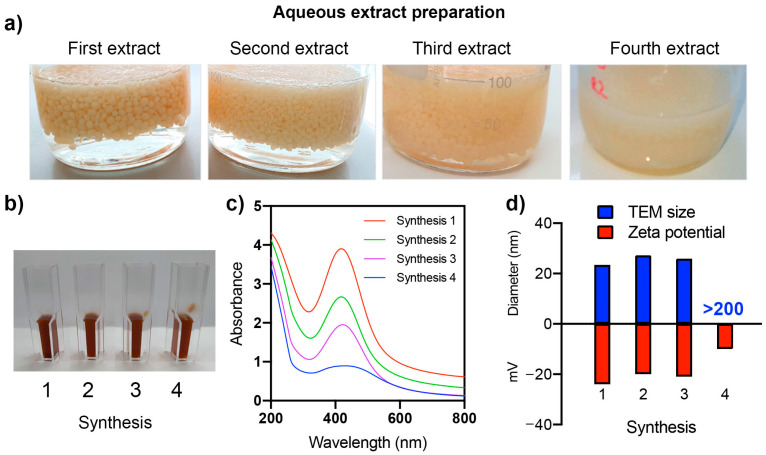
Multiple preparations of aqueous extracts from the same chitosan fungal beads (**a**). The appearance of dispersion (**b**), UV−vis spectra (**c**), and TEM size and zeta potential (**d**) of synthesized nanoparticles using chitosan fungal beads extracts and AgNO_3_ are shown.

The extract preparation and synthesis of nanoparticles were performed four times using the same chitosan fungal beads. The chitosan fungal beads remained stable until the third extraction cycle, but in the fourth cycle, they dissolved and lost their shape (Figure 3a). Figure 3b,c shows the images and UV-vis spectra of the nanoparticle dispersions synthesized in each cycle. The synthesized nanoparticles exhibited a characteristic absorption peak at 420 nm due to the surface plasmon resonance (SPR) of the nano-sized Ag. After each synthesis cycle, a decrease in light absorbance was observed (as shown in Figure 3c). This decrease in absorbance may indicate a reduction in the synthesis yield, which could be caused by the depletion of the compounds present in the chitosan fungal beads that are involved in the synthesis of Ag NPs [18]. Multiple extractions from the same biomass may lead to a decrease in the concentration and availability of these active compounds in the extracts, resulting in a decline in the Ag NP yield.

The dispersions of the fourth synthesis cycle had a flattened UV-vis spectrum with low absorbance at 420 nm. Until the third synthesis, no significant differences in the size (25–27 nm) or zeta potential (from −27 to −20 mV) of the nanoparticles were observed (Figure 3d). The nanoparticles obtained in the fourth synthesis showed a larger size of over 210 nm and a zeta potential of −10 mV.

**Figure 4:**
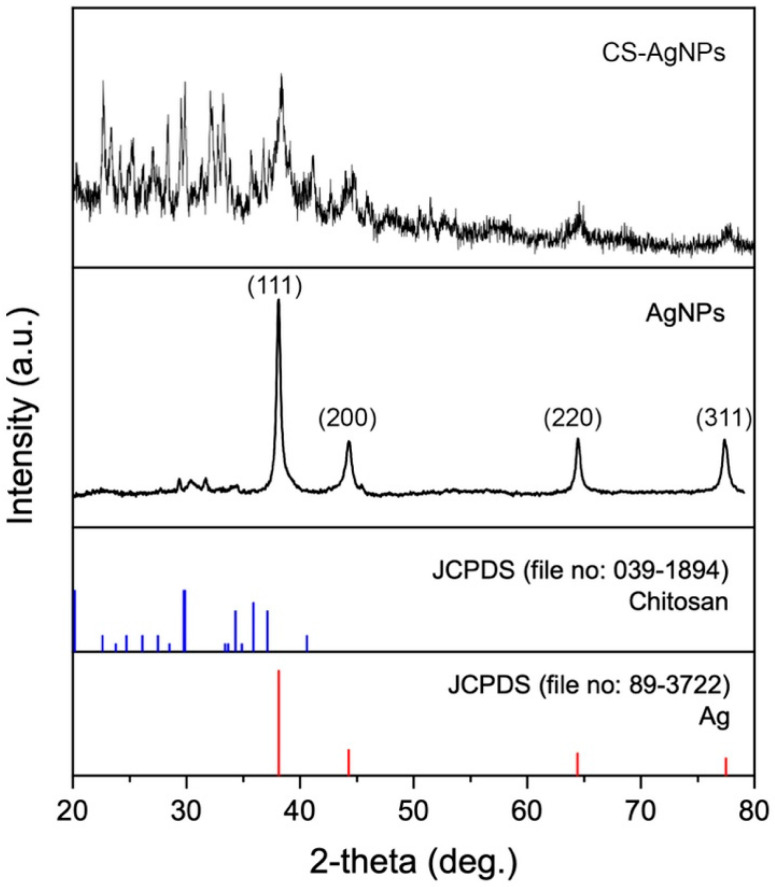
XRD patterns of CS-Ag NPs and Ag NPs synthesized using chitosan-immobilized biomass and an aqueous extract of unencapsulated fungal biomass of *S. hirsutum*.

Figure 4 shows the XRD pattern of nanoparticles synthesized using an aqueous extract of unencapsulated fungal biomass (as control synthesis) and chitosan fungal beads of *S. hirsutum*. The XRD pattern of Ag NPs synthesized with extracts of unencapsulated fungal biomass showed characteristic peaks at 38.11°, 44.27°, 64.42°, and 77.48°, corresponding to (111), (200), (220), and (311) for the face-centered cubic structure of metallic silver. The peaks near 30° could correspond to the presence of organic compounds in the particles that can act as capping agents. The capping agents can sometimes result in electrostatic and steric stabilization of nanoparticles [19].

The XRD pattern of nanoparticles synthesized using the aqueous extract of chitosan fungal beads showed that they were composed of Ag^0^ and chitosan. This suggests that during the extract preparation using chitosan fungal beads, small amounts of chitosan can be released and stabilize the Ag NPs in the synthesis, resulting in the formation of chitosan-silver nanoparticles (CS-Ag NPs).

**Figure 5:**
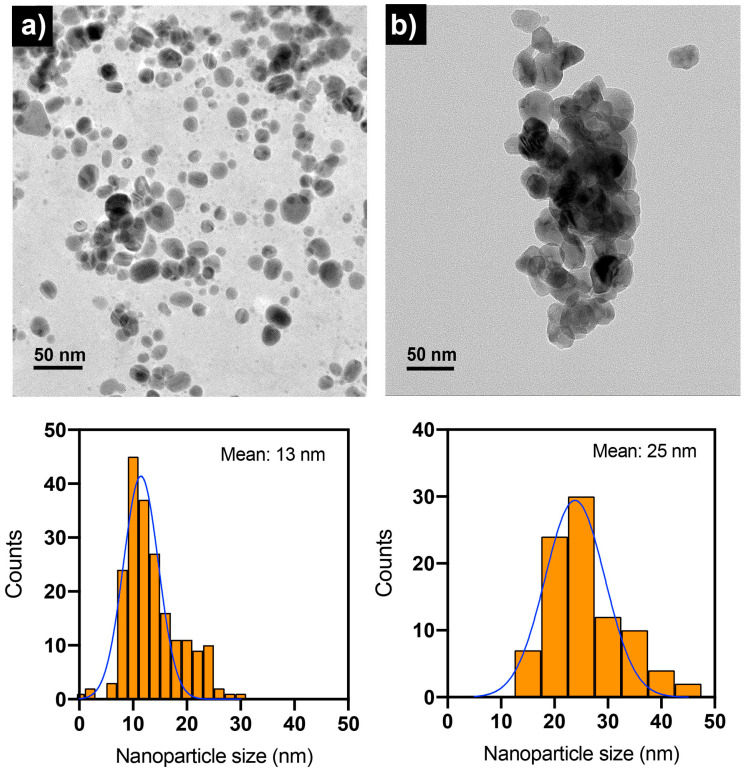
TEM images and nanoparticle size distribution histograms of Ag NPs (**a**) and CS-Ag NPs (**b**) synthesized using extracts of unencapsulated fungal biomass and chitosan-immobilized biomass of *S. hirsutum*, respectively. The “as-synthesized” nanoparticle suspensions were employed in TEM analysis with a pH near 10.

**Figure 6:**
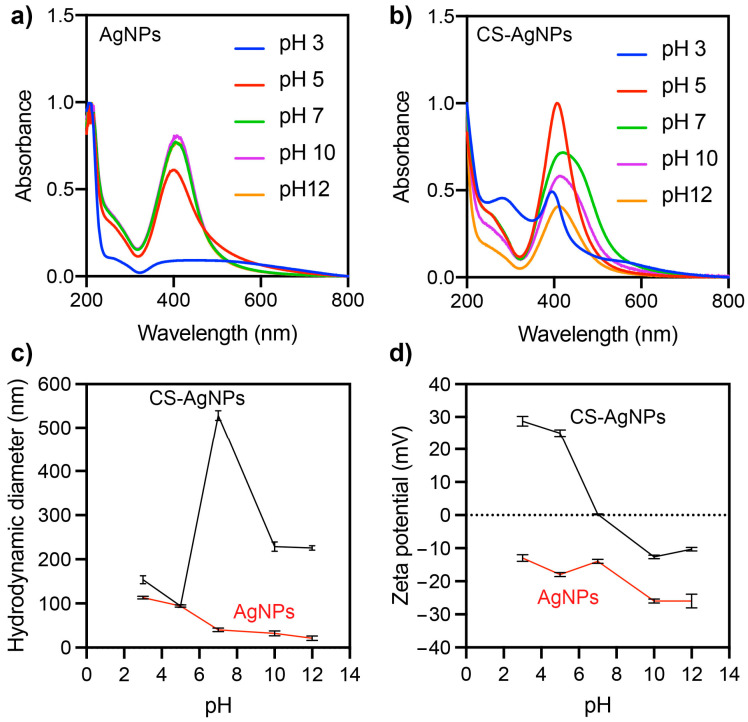
Effect of pH on the UV-vis (**a**,**b**), hydrodynamic diameter (**c**), and zeta potential (**d**) of synthesized Ag NPs and CS-Ag NPs.

TEM was used to examine the size distribution and shape of Ag NPs and CS-Ag NPs as synthesized (Figure 5). The synthesized nanoparticles using either extract from unencapsulated fungal biomass or chitosan fungal beads had a quasi-spherical shape. The size histograms of the nanoparticles displayed a normal distribution pattern for both. The Ag NPs were smaller, with a size of 13 nm compared with the CS-Ag NPs at 25 nm. It was also observed that the CS-Ag NPs were less stable than Ag NPs because they were prone to forming aggregates (Figure 5b). The samples of the nanoparticles analyzed by TEM in their “as-synthesized” state exhibited a pH near 12. However, as depicted in Figure 6, the stability of the CS-Ag NPs was enhanced when the pH fell within a range of 4–6, as evidenced by a higher absolute zeta potential value. In order to investigate the role of chitosan in the synthesized CS-Ag NPs using a chitosan fungal biomass, the antimicrobial activity of these nanoparticles was compared to those of Ag NPs synthesized using aqueous extract of a fungal biomass not encapsulated in chitosan. This allowed for a direct comparison between the two synthesis methods and the potential influence of chitosan on the properties of the resulting nanoparticles. By conducting these assays, it was possible to determine the impact of chitosan on the antimicrobial activity and other characteristics of the CS-Ag NPs.

### 2.3. Effects of pH on the Stability of Ag NPs and CS-Ag NPs

The pH of the dispersion can significantly affect the properties of nanoparticles, such as the solubility, stability, aggregation, and surface charge. Therefore, TEM showed that CS-Ag NPs have a tendency to aggregate. To improve their stability, the impact of pH modification on the dispersion was evaluated. This effect was evaluated on the UV-vis spectra, hydrodynamic size, and zeta potential of Ag NP and CS-Ag NP dispersions (Figure 6). It was observed that the hydrodynamic size and UV-vis absorption spectrum of the Ag NPs were not significantly affected in the pH range between 7 and 12 (Figure 6a). However, at a pH of five, there was an increase in the hydrodynamic size and a decrease in the SPR of the Ag NPs. At a pH of three, the SPR disappeared completely, and the hydrodynamic size of the Ag NPs increased. The zeta potential of the Ag NPs remained at negative values over the pH range evaluated (from 3 to 12).

The lowest values of the zeta potential of the Ag NPs were observed at pH levels of 10–12, while an increase was observed at pH levels below 7. Therefore, the Ag NPs would exhibit greater stability and monodispersibility at alkaline pH values above 10. Monodispersibility refers to the uniformity in particle size within a batch of nanoparticles, and stability refers to the ability of the nanoparticles to maintain their size and shape over time. High absolute zeta potential values (higher than +25 mV or lower than −25 mV) can contribute to the stability and monodispersibility of nanoparticles by helping to prevent aggregation [20]. More significant pH effects were observed for CS-Ag NPs than Ag NPs. In a pH range from 7 to 12, a similarly shaped absorption spectrum was observed, but as the pH approached a neutral value, the absorbance of the SPR increased. At a pH of 5, a narrower SPR with greater absorbance was observed. At a pH of 3, a significant decrease in the SPR and the appearance of a peak close to 280 nm were observed. Chitosan typically absorbs UV light at wavelengths between 200 and 300 nm, with the maximum absorption occurring at around 280 nm [21]. This UV absorption is due to the presence of conjugated double bonds in the chitosan molecule, which can absorb UV light and produce an electronic transition. Therefore, the peak at 280 nm can be attributed to the destabilization and subsequent solubilization of chitosan from the surface of the nanoparticles. The hydrodynamic size of the CS-Ag NPs also varied with respect to the pH of the solution. Smaller sizes could be observed at a pH of five and an increase at neutral and alkaline pH levels. The zeta potential of the CS-Ag NPs was also affected by the pH of the solution. CS-Ag NPs had a negative zeta potential at alkaline pH levels, a positive zeta potential (27 mV) at acidic pH levels, and an isoelectric point at a neutral pH level. One potential explanation for the observed effect of the pH level on the zeta potential of CS-Ag NPs is the protonation or lack of protonation of the amino groups present on the chitosan molecule within the nanoparticles. Chitosan is a polysaccharide composed of randomly distributed D-glucosamine and N-acetyl-D-glucosamine, which are linked by β-(1→4) glycosidic bonds. Commercially, chitosan is produced by the deacetylation of chitin. The D-glucosamine units contain amino groups that can become protonated or unprotonated, depending on the pH of the environment. At pH values below the pKa of the amino groups (typically between 6.0 and 6.5) [22], the amino groups on the chitosan molecule became protonated and positively charged, leading to a positive zeta potential for the CS-Ag NPs. At pH values above the pKa, the amino groups on the chitosan molecule were unprotonated and neutral, leading to a negative zeta potential for the CS-Ag NPs. These results indicate that CS-Ag NPs are more stable and monodispersed at a pH of five. The pH of the solution had a significant effect on the properties of CS-Ag NPs. These findings demonstrate the importance of the pH level in controlling the properties of CS-Ag NPs.

### 2.4. Antimicrobial Activity of Ag NPs and CS-Ag NPs

The observed differences in the properties of Ag NPs and CS-Ag NPs, especially when their pH levels were modified, led to the evaluation of their antimicrobial activity against three human pathogens and one plant pathogen. The human pathogens tested included *Escherichia coli*, *Staphylococcus aureus*, and *Candida albicans*. *E. coli* is a gram-negative bacterium commonly found in the human gut and is generally harmless. However, some strains of *E. coli* can cause food poisoning or urinary tract infections [23]. *S. aureus* is a gram-positive bacteria found on the skin and in the nose for many people. It is generally harmless but can cause infections if it enters the body through a cut or wound. *S. aureus* infections can range from mild skin infections to more severe infections such as pneumonia or bone infections [24]. *C. albicans* is a yeast commonly found in the human body, particularly in the mouth, throat, and gut. It is usually harmless but can cause infections if it overgrows in certain body areas, such as the mouth or genitals [25]. The plant pathogen tested was *P. syringae*, a highly destructive phytopathogen that is found throughout the world. It is responsible for causing bacterial canker disease, which can attack the stones of developing fruit and severely damage trees. This can result in reduced yields and significant economic losses for farmers and agricultural industries. *P. syringae* is particularly dangerous because it can infect many plant species, including important crops and fruit trees [3]. The antimicrobial activity of the CS-Ag NPs was tested, including a treatment adjusted to a pH of 5, where they were more stable and smaller. This allowed for a more accurate evaluation of their antimicrobial activity at the optimal pH for their stability and hydrodynamic size. The minimum inhibitory concentration (MIC) of the Ag NPs and CS-Ag NPs against the pathogens is shown in Table 1. The MIC is the lowest antimicrobial agent concentration that can inhibit a microorganism. The results of this study showed that CS-Ag NPs had stronger antimicrobial activity than Ag NPs against the gram-negative and yeast pathogens tested. The CS-Ag NPs had a MIC of 1.5 µg/mL against *P. syringae* at pH levels of 5 and 10. This was 27-fold lower than the MIC of the Ag NPs (40 µg/mL), indicating that CS-Ag NPs are more effective against this pathogen. The antibiotic control, using tetracycline, showed a MIC of 1 µg/mL against *P. syringae*. In the case of *E. coli*, the MIC for the CS-Ag NPs was 1.6 µg/mL at a pH of 5 and 6.2 µg/mL at a pH of 10, reaching a value of 7.8-fold lower than the MIC of the Ag NPs (12.5 µg/mL). The Ag NPs and CS-Ag NPs showed the same MIC of 3.1 µg/mL against *S. aureus*. The antibiotic control, using ceftazidime, showed an MIC of 0.25 and 8 µg/mL against *E. coli* and *S. aureus*, respectively. The MIC of the CS-Ag NPs against *C. albicans* was found to be 16 µg/mL at a pH of 5 and 4 µg/mL at a pH of 10. The MIC value at a pH of 10 was fourfold lower than the MIC of the Ag NPs, indicating that the CS-Ag NPs were more effective than Ag NPs against this pathogen. Contrary to the observed results of antimicrobial activity against bacteria, the MIC of the CS-Ag NPs was found to be higher at a pH of 5 than at a pH of 10 when tested against *C. albicans*. This suggests that the compound may be less effective at inhibiting the growth of *C. albicans* at lower pH levels. Ahamad et al. (2022) reported that the minimum inhibitory concentration (MIC) value of the biogenic Ag NPs with a size range of 11–15 nm was 12.5 µg/mL against *C. albicans* [26]. Another study reported higher MIC values for Ag NPs against *C. albicans*, ranging from 1080 to 580 µg/mL [27].

Our results indicate that chitosan enhances the antimicrobial activity of Ag NPs under in vitro conditions, especially against gram-negative bacteria and yeast. Shahryari et al. (2020) conducted a study to evaluate the antimicrobial activity of Ag NPs and CS-Ag NPs against a strain of *P. syringae*. Their results indicated that both Ag NPs and CS-Ag NPs are effective at inhibiting the growth of *P. syringae*, with MICs of 12 and 4 µg/mL, respectively [3]. Sherif et al. (2017) synthesized a chitosan-silver nanocomposite through a chemical method. They reported its antimicrobial activity against bacterial species, including *S. aureus*, *Pseudomonas aeruginosa*, and *E. coli*. The MIC of the chitosan-silver nanocomposite was 220 µg/mL [28]. The antimicrobial activity of chitosan and silver can be enhanced due to the cumulative effect between them in the form of CS-Ag NPs. Chitosan and its derivatives contain polycationic amines that can interact with the negatively charged components on the surface of bacterial cells, leading to disruption of the cell membrane, the leakage of cytoplasmic components, and the death of the cell [3]. The molar percentage of glucosamines found in chitosan is determined by the degree of deacetylation. This varies depending on the method used to produce commercial chitosan, with deacetylation degrees ranging from 60 to 100% [29]. In this study, the chitosan used in the preparation of chitosan fungal beads had a deacetylation degree of ≥75%. On the other hand, the mechanism by which Ag NPs exert their antimicrobial activity has yet to be fully understood. Still, several mechanisms have been proposed, including interference with the respiratory chain of bacteria and fungi, leading to a reduction in ATP production and cell death, binding to and inactivating enzymes involved in DNA synthesis, replication, and repair, leading to DNA damage, interaction with the cell membrane of bacteria and fungi, causing changes in membrane permeability and integrity, and releasing silver ions (Ag^+^) which can interact with proteins and enzymes in the cell, leading to cell death [30,31,32,33]. Therefore, the cumulative effect observed in the antimicrobial activity of CS-Ag NPs can be attributed to the combined action of chitosan to disrupt the cell membrane and metallic silver to inhibit cellular metabolism [34]. In addition, the small size of the nanoparticles allows them to enter cells easily and interact with cellular components, which may further enhance their antimicrobial activity [8]. The pH of the solution has a significant effect on the antimicrobial activity of CS-Ag NPs against certain microorganisms. In this study, the effect of the pH level was more pronounced in the activity of CS-Ag NPs against the gram-negative bacterium *E. coli* and *P. syringae*. This can be attributed to the positive zeta potential and chitosan protonation of CS-Ag NPs at a pH of five, which promoted their adsorption to the negatively charged cell wall of microorganisms. This could increase their antimicrobial activity by increasing their contact with the microorganisms and enhancing their ability to disrupt their cell membranes. However, the chitosan content and pH appeared to have no significant effect on the activity of CS-Ag NPs against gram-positive bacteria, as was observed in the MIC against *S. aureus*. The gram-positive bacteria have thick peptidoglycan cell walls, while gram-negative bacteria have a thinner peptidoglycan cell wall surrounded by an outer membrane containing lipopolysaccharides [30]. The thicker peptidoglycan cell wall of gram-positive bacteria makes them more permeable to nanoparticles. Therefore, even though gram-positive bacteria also have a negative surface charge, the mechanisms of Ag NPs against gram-positive bacteria may not be dependent on electrostatic interactions. Thus, the antimicrobial activity of Ag NPs against gram-positive bacteria is thought to involve a combination of physical damage, oxidative stress, and interference with bacterial metabolism, ultimately leading to cell death. Our findings suggest that CS-Ag NPs have the potential to be a promising alternative to traditional antimicrobial agents for treating a wide range of infections, including those related to wound healing, skin infections, dental care, and the food industry. However, as chitosan is derived from crustacean shells, it is important to note that there is a possibility that these nanoparticles may cause allergic reactions in some individuals. Therefore, it is crucial to conduct further research to evaluate the potential allergenic effects of CS-Ag NPs before they can be widely adopted. One way to eliminate the potential for allergic reactions associated with chitosan derived from crustacean shells is to use chitosan sourced from non-allergenic sources such as fungi.

## 3. Materials and Methods

### 3.1. Materials

Potato dextrose agar (PDA), glucose, potato peptone, yeast extract, silver nitrate (AgNO_3_), and sodium hydroxide (NaOH) were purchased from Merck (Darmstadt, Germany). Chitosan, sodium tripolyphosphate, Muller–Hinton broth, RPMI 1640 media, ceftazidime, and amphotericin B were obtained from Sigma-Aldrich (Darmstadt, Germany).

### 3.2. Microorganism

The white-rot fungus *Stereum hirsutum* (code CCCT22.02) was sourced from the environmental biotechnology laboratory at the Universidad de La Frontera in Chile. The fungus was carefully maintained in PDA plates at a temperature of 4 °C and regularly subcultured to ensure its viability in experiments. This strain of fungus has previously been shown to exhibit promising properties for use in the synthesis of Ag NPs [17].

### 3.3. Fungal Biomass Obtention

To obtain fungal biomass, 5 6-mm-in-diameter agar-mycelia disks from 7-day-old *S. hirsutum* culture plates were transferred to a 250 mL Erlenmeyer flask containing 100 mL of liquid growth medium consisting of 15 g/L glucose, 5 g/L potato peptone, and 2.5 g/L yeast extract. The flasks were incubated at 25 °C for 14 days under static conditions to allow the formation of fungal mycelium. After incubation, the mycelium was collected by vacuum filtration using Whatman paper No. 1 and washed with deionized water to remove any residual culture medium that could interfere with the synthesis process. Parallel experiments were conducted to obtain an aqueous extract of unencapsulated fungal biomass for use in the synthesis of silver nanoparticles (AgNPs) and to encapsulate the fungal biomass in chitosan beads for use in the synthesis of chitosan-silver nanoparticles (CS-Ag NPs).

### 3.4. Preparation of Chitosan Fungal Beads

To prepare chitosan fungal beads, the washed fungal biomass of *S. hirsutum* (600 mg dry weight) was homogenized in a sterile blender for 2 min and then centrifuged at 4100 rpm for 10 min to obtain a precipitate. The precipitate was mixed with 50 mL of 2% chitosan (low molecular weight: 50–190 kDa; deacetylation degree: >75%) and encapsulated using a B-390 encapsulator (BÜCHI Labortechnik AG, Flawil, Switzerland) equipped with a concentric nozzle of 1 mm and operated under a vibration frequency of 120 Hz and air pressure of 72 mBar. The dispersed droplets were hardened by treating them with 1% (*w/v*) sodium tripolyphosphate while stirring magnetically for 24 h to form chitosan-immobilized beads. Chitosan beads were produced under the same conditions but without including fungal biomass. The average size of the resulting chitosan fungal beads was determined using ImageJ software, and their morphology was characterized using scanning electron microscopy (SEM) and confocal laser scanning microscopy (CLSM) with a BD Cell Viability Kit to evaluate the structure of the beads.

### 3.5. Synthesis of Nanoparticles Using Extract of Chitosan Fungal Beads

The chitosan fungal beads (~750–800 beads) were transferred to 50 mL of deionized water and incubated for 24 h at 25 °C and 100 rpm. The chitosan fungal beads were then removed by vacuum filtration using Whatman N°1 filter paper, and the resulting 50 mL of aqueous extract was collected and reused four times. Additionally, an aqueous extract was prepared using chitosan beads that did not contain any fungal biomass as a control experiment. To synthesize the CS-Ag NPs, the aqueous extract was adjusted to a pH of 12 using 3 M NaOH, and then 100 mM of AgNO_3_ solution was added to obtain a concentration of 3 mM in the synthesis mixture. The mixture was incubated for 48 h under constant agitation at 150 rpm. The resulting particles were frozen at −80 °C, freeze-dried, and then stored for further experiments.

### 3.6. Synthesis of Nanoparticles Using Extract of Unencapsulated Fungal Biomass

To synthesize Ag NPs, a method developed by Hermosilla et al. (2022) was followed using an aqueous extract of unencapsulated fungal biomass obtained from *S. hirsutum* culture. The extract was prepared by transferring fungal biomass (800 mg of dry matter) to a 100 mL Erlenmeyer flask containing 50 mL of deionized water and incubating the mixture in an orbital shaker at 100 rpm and 25 °C for 24 h. The supernatant was collected by vacuum filtration, and the fungal biomass was discarded. The Ag NPs were synthesized in a mixture containing 50 mL of the extract fraction and 3 mM AgNO_3_ as the precursor salt, which was kept under magnetic agitation at room temperature. The pH was adjusted to 12 by adding drops of 4 M NaOH, and the nanoparticle dispersions were shaken at 150 rpm for 7 days before characterization. The resulting particles were frozen at −80 degrees Celsius, freeze-dried, and then stored for further experiments.

### 3.7. Characterization of Synthesized Nanoparticles

The synthesized particles were characterized by using various techniques to assess their properties and determine their potential applications. Their hydrodynamic diameter and surface charge (zeta potential) were measured using a Zetasizer Nano ZS90 (Malvern Instruments, Inc., Worcestershire, UK), while their optical properties were analyzed using UV-visible absorption spectroscopy (Thermo Scientific EvolutionTM 60S, Kyoto, Japan) in the range of 200–800 nm. Fourier transform infrared spectroscopy (FTIR) was performed to identify the functional groups in the synthesized nanomaterials, with lyophilized samples analyzed using a Cary 630 FTIR instrument (Agilent Technologies, Santa Clara, CA, USA). X-ray diffraction (XRD) analysis was carried out using a Rigaku Smartlab X-ray diffractometer (Rigaku Corporation, Tokyo, Japan) operating at room temperature, with CuKα radiation in the 2θ range from 10 to 80 K. The morphology and particle size distribution of the nanomaterials were determined using transmission electron microscopy (TEM) with a Libra 120 Zeiss instrument operating at 120,000 kV. These techniques allowed for a comprehensive characterization of the synthesized particles.

### 3.8. Antimicrobial Activity of Ag NPs and CS-Ag NPs against Human Pathogen Bacteria

The minimum inhibitory concentration (MIC) against the human pathogen bacteria *E. coli* ATCC 25922 and *S. aureus* ATCC 25923 was assessed using the synthesized nanoparticles. In this experiment, nanoparticle samples were prepared by sonicating them and then diluting them in a range of concentrations from 0.04 to 100 µg/mL in Muller–Hinton broth. These diluted samples were then distributed in 96-well microwell plates at a final volume of 100 µL and tested in duplicate. Ceftazidime (CAZ) was used as a positive control and was similarly diluted in a range of concentrations from 0.03 to 32 µg/mL at a final volume of 100 µL. Both the nanoparticle samples and the CAZ control were tested against two pathogenic strains that were inoculated in each microwell plate at an optical density (OD) of 0.1 McFarland. The microwell plates were homogenized by mixing for 10 min and then incubated at 37 °C for 20 h. The procedures and interpretation of the antibiotic susceptibility results were carried out according to the Clinical and Laboratory Standards Institute (CLSI) guidelines from 2016. A negative control using culture broth was also included in the experiment.

### 3.9. Antimicrobial Activity of Ag NPs and CS-Ag NPs against Human Pathogen Yeast

The MIC of synthesized nanoparticles against the yeast strain *Candida albicans* ATCC90028 was assessed. Nanoparticle samples were prepared by sonicating them and then diluting them in a range of concentrations from 0.05 to 100 µg/mL in RPMI 1640 media supplemented with L-glutamine. These diluted samples were distributed in 96-well microwell plates at a final volume of 100 µL. Amphotericin B was used as a positive control and was similarly diluted in a range of concentrations from 0.03 to 32 µg/mL at a final volume of 100 µL. The pathogenic strain was inoculated in each microwell plate at an optical density (OD) of 0.5 McFarland and later diluted at a 1:1000 ratio in RPMI 1640. A final volume of 100 µL of the diluted strain was then added to each well, and the microwell plates were incubated at 35° for 48 h. The procedures and interpretation of the antibiotic susceptibility results were carried out according to the Clinical and Laboratory Standards Institute (CLSI) M27-A3 guidelines. A negative control using culture media was also included in the experiment.

### 3.10. Antimicrobial Activity of Ag NPs and CS-Ag NPs against Phytopathogen Bacterium

The antimicrobial activity of the synthesized nanoparticles was tested against the phytopathogenic bacterium *P. syringae* CCCT22.03 strain, which was obtained from the Chilean Collection of Cultures of the University of La Frontera (CCCT-UFRO) and grown according to standard guidelines (CLSI 2012). First, 150 µL of the bacterial strain was inoculated in 10 mL of sterile Mueller–Hinton culture medium and incubated at 28 degrees Celsius and 150 rpm until the optical density at 600 nm reached ~1–2 × 10^6^ CFU/mL. Aliquots of 20 µL of the bacterial culture were then transferred to a 96-well plate to perform the antimicrobial test, with nanomaterial concentrations ranging from 0.5 to 50 µg/mL. All treatments were performed in triplicate, including control treatments of nanomaterials without bacterial inoculum and the bacterium without nanomaterials. The plate was incubated for 24 h at 28 °C under static conditions, and then the optical density (OD_600nm_) was measured using an Epoch Spectrophotometer System (BioTek Instrument Inc., Winooski, VT, USA). Antibiotic susceptibility results and interpretations were carried out according to CLSI 2016 guidelines.

## 4. Conclusions

Chitosan fungal beads were found to be an effective and reusable source of fungal compounds for synthesizing CS-Ag NPs. By using these beads, low amounts of chitosan can be obtained in the extract, which stabilize the CS-Ag NPs. Additionally, chitosan provides a positively charged surface on the nanoparticles, resulting in enhanced electrostatic interactions with negatively charged microorganisms and increased antimicrobial activity. This method could be used to develop a continuous process for synthesizing CS-Ag NP nanoparticles at a large scale. This could make the production of CS-Ag NPs more efficient and cost-effective. The results of this study showed that CS-Ag NPs have strong antimicrobial activity against a variety of human and plant pathogens. Therefore, CS-Ag NPs may be a promising alternative to conventional antimicrobial agents for treating infections and controlling plant diseases. Further research is necessary to confirm the efficacy of CS-Ag NPs as antimicrobial agents in various applications through stability and storage tests and in vivo antimicrobial testing as well as to evaluate their safety through in vivo testing to determine potential toxicity or allergic reactions.

## Figures and Tables

**Table 1:** The minimum inhibitory concentration of synthesized nanomaterials against phytopathogen and human pathogen microorganisms.

Strains		Minimum Inhibitory Concentration (MIC µg mL^−1^)		
	Antibiotic Control	Ag NPs (pH 10)	CS-Ag NPs (pH 10)	CS-Ag NPs (pH 5)
*P. syringae* CCCT22.02 ^a^	1.0 TC	40.0	1.5 (27-fold)	1.5 (27-fold)
*E. coli* ATCC 25922 ^a^	0.25 CAZ	12.5	6.2 (2-fold)	1.6 (7.8-fold)
*S. aureus* ATCC 25923 ^b^	8.0 CAZ	3.1	3.1	3.1
*C. albicans* ATCC90028 ^c^	0.03 ANF	16	4 (4-fold)	16

^a^ Gram-negative bacterium. ^b^ Gram-positive bacterium. ^c^ Yeast. The number of reduction folds in minimum inhibitory concentration (MIC) compared with Ag NPs is in parenthesis. CAZ: ceftazidime; STM: streptomycin; TC: tetracycline; ANF: amphotericin.

## Data Availability

No applicable.
